# Alignment of Common Wheat and Other Grass Genomes Establishes a Comparative Genomics Research Platform

**DOI:** 10.3389/fpls.2017.01480

**Published:** 2017-08-30

**Authors:** Sangrong Sun, Jinpeng Wang, Jigao Yu, Fanbo Meng, Ruiyan Xia, Li Wang, Zhenyi Wang, Weina Ge, Xiaojian Liu, Yuxian Li, Yinzhe Liu, Nanshan Yang, Xiyin Wang

**Affiliations:** ^1^School of Life Sciences, North China University of Science and Technology Tangshan, China; ^2^Center for Genomics and Computational Biology, North China University of Science and Technology Tangshan, China

**Keywords:** common wheat, grass, genome, gene collinearity, polyploidization

## Abstract

Grass genomes are complicated structures as they share a common tetraploidization, and particular genomes have been further affected by extra polyploidizations. These events and the following genomic re-patternings have resulted in a complex, interweaving gene homology both within a genome, and between genomes. Accurately deciphering the structure of these complicated plant genomes would help us better understand their compositional and functional evolution at multiple scales. Here, we build on our previous research by performing a hierarchical alignment of the common wheat genome vis-à-vis eight other sequenced grass genomes with most up-to-date assemblies, and annotations. With this data, we constructed a list of the homologous genes, and then, in a layer-by-layer process, separated their orthology, and paralogy that were established by speciations and recursive polyploidizations, respectively. Compared with the other grasses, the far fewer collinear outparalogous genes within each of three subgenomes of common wheat suggest that homoeologous recombination, and genomic fractionation should have occurred after its formation. In sum, this work contributes to the establishment of an important and timely comparative genomics platform for researchers in the grass community and possibly beyond. Homologous gene list can be found in Supplemental material.

## Introduction

Poaceae is a large family of monocotyledonous flowering plants, commonly known as grasses, consisting of more than 600 genera and more than 10,000 species. It is recognized as the most economically important plant family, as it accounts for 70% of planted crops, thus providing a key food source for humans (Kellogg, 1998; Gaut, 2002; Paterson et al., 2004). In recent years the genomes of several grasses have been sequenced: namely, of rice (*Oryza sativa*), sorghum (*Sorghum bicolor*), maize (*Zea mays*), foxtail millet (*Setaria italica*), purple false brome (*Brachypodium distachyon*), barley (*Hordeum vulgare*), and two diploid wheat species (wheat D genome of *Aegilops tauschii*, wheat A genome of *Triticum urartu*) (International Rice Genome Sequencing, 2005; Paterson et al., 2009; Schnable et al., 2009; International Brachypodium, 2010; Bennetzen et al., 2012; Mayer et al., 2012; Zhang et al., 2012; Du et al., 2017; Mascher et al., 2017). In addition, the genome sequences of common wheat have also been deciphered (Brenchley et al., 2012; Berkman et al., 2013; Jia et al., 2013; Ling et al., 2013; Mayer et al., 2014; Middleton et al., 2014; Clavijo et al., 2017; Zimin et al., 2017). These sequencing efforts have provided valuable data for advancing biological and breeding research in plants.

Recursive polyploidizations have contributed to the evolution of grasses. The sequencing of the rice genome revealed a grass-common tetraploidization—or whole-genome duplication (WGD) event—that occurred 100 million years ago (Paterson et al., 2004; Wang et al., 2005). This event might have played a major role in promoting the speciation of new grasses to form the large monocot family that exists today. Since then, further polyploidizations continued to occur in this family, including one that likely contributed directly to the formation of maize, and two sequential ones that contributed to the origin of common wheat (*Triticum aestivum*). The latter arose through hybridization of the wheat genome A (*Triticum monococum*) with the genome B, which afterward hybridized with genome D (*A. tauschii*). Recursive polyploidizations greatly complicate the structure of plant genomes, and this process produces large numbers of duplicated genes even after widespread post-polyploidy gene losses occur. Nonetheless, these duplicated genes arising from polyploidization are an important evolutionary driving force, one that has exerted its biological effects for millions of years.

An accurate alignment of multiple genomes is critical to better understanding their structures, to reveal homologous genes, and to infer how evolutionary events actually unfolded. By using the rice genome as a reference, and an by examining their gene collinearity, XW and JW were able to successfully align several sequenced grass genomes (Wang X. et al., 2015b), but this was done before the genome of common wheat had become available. Nevertheless, that study provided insight into the genomic changes after the divergence in grasses, and it helped re-date key events during the evolution of the Poaceae family. The identified homologous genes were well-related to each recursive polyploidization and to each speciation event, making it possible to hierarchically distinguish the paralogous and orthologous genes. This genetic information is valuable for understanding the genome structure formation and its overall changes, and in particular for clarifying cases of gene divergence, and phylogeny. During this alignment process, the rice genome served as a reference because it is well-sequenced and assembled and has conserved its genome structure, and gene evolution (Salse et al., 2009; Wang X. et al., 2015b).

Here, we build on this prior work to take advantage of the now-available genome of common wheat, by adding it to the previously constructed multiple-genome alignment of grass species. Although only one new species is added here, it has three subgenomes, and its inclusion thus required considerable effort to achieve. Besides, we have involved the most updated assemblies and/or annotations of rice, barley, and other grasses in the present analysis. The present effort aims at producing a list of homologous (paralogous and orthologous) genes, related to different polyploidizations, and speciations, characterizing genomic instability of common wheat, and contributing to establishing the grass comparative genomics platform.

## Materials and methods

### Materials

Grass genomes and their gene annotations for each species were downloaded (Supplementary Table 1). Then the data were preprocessed.

### Analysis of genomic homology

To obtain the gene collinear homologs, we used the BLASTP to search for the potential anchors (*E* < 1e-5; top five matches) between every possible pair of chromosomes within the 9 grasses, and between every possible species pair. Based on these results, dot-plots were drawn by using the Perl scripts to perform an illustrative comparison of the genomes to better understand their structures. By using the software MCSCAN (Tang et al., 2008) and CollinearScan (Wang et al., 2006), we identified those homologous blocks containing collinear genes within a genome and between different genomes (maximal searching gap ≤ 50 genes; *P* < 0.05). By characterizing the homologous sequence similarities, as measured by both the collinear gene number and sequence identity, we then distinguished the paralogous and orthologous genes among them, as detailed previously (Wang X. et al., 2015b).

## Results

### Pairwise alignment of the genomes

By inferring gene collinearity, we performed a whole-genome multiple alignment for common wheat vis-à-vis the sequenced grass genomes of rice, purple false brome, barley, foxtail millet, sorghum, maize, and two diploid wheat species (Supplementary Table 2). The duplicated genes produced by the grass-common tetraploidization (GCT) and the maize-specific tetraploidizaton (MST) were thus obtained. Based on the derived intraspecific and interspecific collinear gene information, we constructed a table of the homologous genes, and their orthologs, and (out) paralogs associated with speciations, and polyploidizations, respectively (Wang X. et al., 2015a,b).

The detailed statistics for the homologous blocks and genes within a plant genome, or between any pair of them, are given in Supplementary Table 3 (wherein any tandem genes were filtered out). Homologous blocks that had more than a certain number of collinear genes were counted to reflect the breakages of genomic homology. The homologous genes in common wheat were further divided into subgroups to show the extent of gene collinearity within each subgenome and between the subgenomes. For example, in the subgenomes A of common wheat, we found 619, 38, 17, and 5 homologous blocks, each with respectively at least 4,10, 20, and 50 collinear gene pairs that contained 4,054, 1,070, 810, and 418 collinear gene pairs in total. In the subgenomes B of common wheat, we found 584, 38, 13, and 8 homologous blocks, each with respectively at least 4,10, 20, and 50 collinear gene pairs that contained 3,806, 986, 651, and 512 collinear gene pairs in total. In the subgenomes D of common wheat, we found 602, 34, 15, and 4 homologous blocks, each with respectively at least 4,10, 20, and 50 collinear gene pairs that contained 3,969, 988, 745, and 379 collinear gene pairs in total. This means nearly one fourth of wheat genes have collinear homology in each subgenome, which is higher than rice, sorghum, and other genomes affected by the GCT, but not maize (28.3%) affected by an extra MST. The finding suggests that the gene-dense regions, often with well-preserved gene collinearity, have been well-assembled.

### Multiple alignments of the genomes

**Figure 1:**
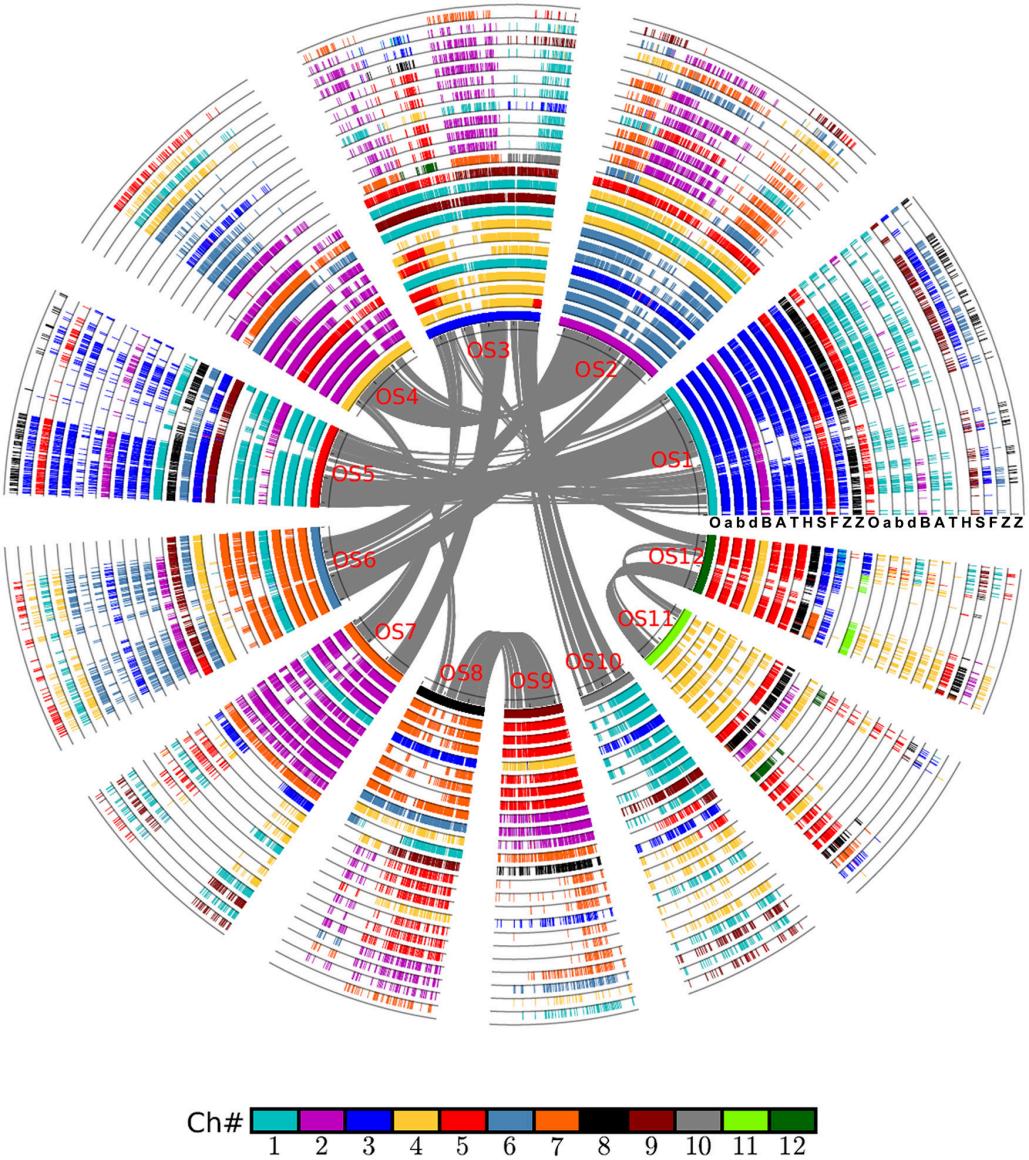
Alignment of the Poaceae chromosomes with rice as reference. Based on gene collinearity, the chromosomes were aligned with rice used as the reference. The whole-genome duplication (WGD) in the common ancestor of these Poaceae plants caused all of them to have at least two circles of chromosomes. An additional lineage-specific diploidization event caused maize to have four chromosomes, and an independent hybridization event caused common wheat to have six such chromosomes. Each grass species has another circle containing additional duplicated regions. Genes are colored according to their correspondence with the rice chromosome. For example, the genes from all Poaceae plants having orthologs on the rice chromosome 1 are given in blue. A, *Aegilops tauschii* (wheat D genome); B, *Brachypodium distachyon*; F, *Setaria italica*; H, *Hordeum vulgare* (barley genome); O, *Oryza sativa* (rice genome); S, *Sorghum bicolor*; T, *Triticum urartu* (wheat A genome); Z, *Zea mays*; a, genome A of *Triticum aestivum* (common wheat); b, genome B of *Triticum aestivum*; d, genome D of *Triticum aestivum*.

**Figure 2:**
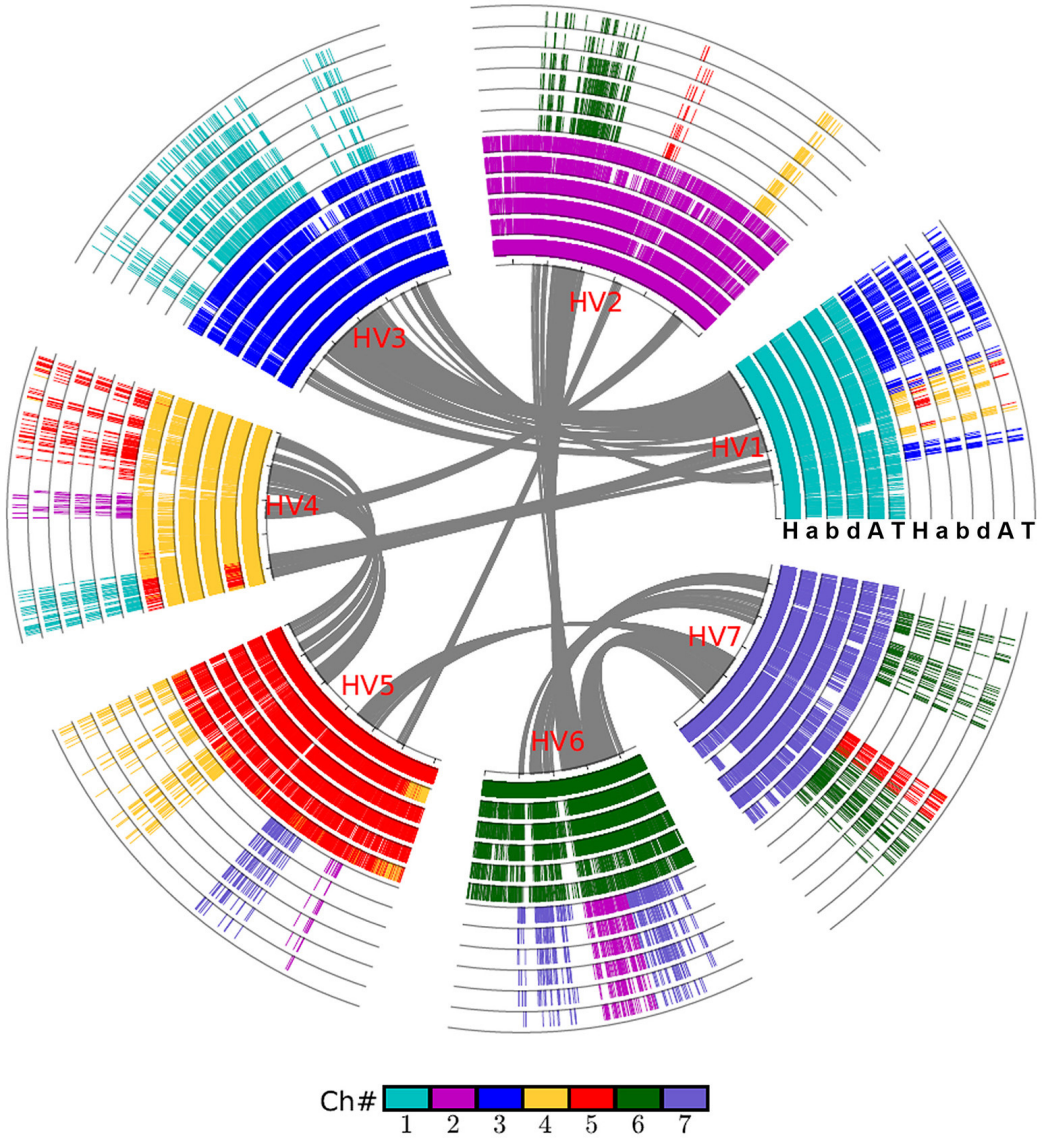
Alignment of the wheat crop chromosomes with barley as reference. Based on gene collinearity, the chromosomes were aligned with barley used as the reference. The whole-genome duplication (WGD) in the common ancestor of these Poaceae plants caused all of them to have at least two concentric circles of chromosomes, and an additional hybridization event caused the common wheat to have six such chromosomes. Each grass species has another concentric circle containing additional duplicated regions. Genes are colored according to their correspondence with the barley chromosome. For example, the genes from all Poaceae plants having orthologs on the barley chromosome 1 are shown in blue. A, *Aegilops tauschii* (wheat D genome); H, *Hordeum vulgare* (barley genome); T, *Triticum urartu* (wheat A genome); a, genome A of *Triticum aestivum* (common wheat); b, genome B of *Triticum aestivum*; d, genome D of *Triticum aestivum*.

By integrating the information on collinear homologs found within the genomes and between them, we were able to first construct an alignment of the grass genomes (Figure 1), and then an alignment of the chromosomes of wheat and its close relatives Figure 2.

The first alignment was built by using rice as a reference. Rice has a well-preserved genome structure that not only closely resembles that of the grass-common ancestor but also has been well-sequenced and assembled. The alignment was done by putting the collinear gene information into a table, wherein the rice gene IDs from 12 of its chromosomes were placed in the first column. However, because of the GCT (experienced together with the other grasses), rice would have to have two columns to contain the duplicated genes, i.e., the GCT paralogs. Similarly, each non-maize grass species would also have two columns, with each being orthologous to one of the rice columns. For maize, however, which experienced an MST, it would have two paralogous columns that corresponded to each one of the two columns in rice (and likewise for each of the other grasses). In the case of common wheat, a hexaploid plant—it is derived from three diploid wheat species—each of the two rice columns would have to have three wheat-orthologous columns. Therefore, for the nine grass genomes studied, the ensuing alignment table had a total of 24 columns in it. Each row of the table contained the collinear homologs, orthologs, or (out) paralogs. For a gene missing from an expected location in a given row, a dot was put in this place to flag this likely gene loss or translocation (deletion/insertion).

Considering their intragenomic homology, the number of paralogous blocks in the different species ranged from 30 to 96 (Table 1), which consisted of 922–6,614 collinear gene pairs (Table 2), and 1,614–9,196 homologous genes (Table 3). Notably, we found 30 homologous blocks involving 2,852 collinear gene pairs, and 4,026 homologous genes in the subgenomes A of common wheat, 31 homologous blocks involving 2,589 collinear gene pairs, and 3,749 homologous genes in the subgenomes B of common wheat, 30 homologous blocks involving 2,778 collinear gene pairs, and 3,975 homologous genes in the subgenomes D of common wheat, most of which were produced by the GCT (Tables 1–3).

**Table 1:** Number of paralogous and orthologous blocks within and among the selected Poaceae genomes.

**Species**		***Oryza sativa***	***Aegilops tauschii***	***Triticum urartu***	***Hordeum vulgare***	***Setaria italica***	***Sorghum bicolor***	***Brachypodium distachyon***	***Zea mays***	***Triticum aestivum***		
										**Genome A**	**Genome B**	**Genome D**
*Oryza sativa*		**59**	433	138	167	145	121	168	307	146	126	152
*Aegilops tauschii*		118	**49**	226	709	264	239	241	276	615	601	610
*Triticum urartu*		72	47	**76**	561	114	97	110	146	160	244	240
*Hordeum vulgare*		87	103	92	**79**	141	136	141	239	464	396	407
*Setaria italica*		109	110	63	78	**54**	78	158	261	119	119	131
*Sorghum bicolor*		94	92	60	73	80	**35**	137	219	96	107	106
*Brachypodium distachyon*		116	81	58	74	85	79	**51**	292	111	122	119
*Zea mays*		207	116	75	123	171	142	152	**96**	217	209	213
*Triticum aestivum*	genome A	67	72	71	108	82	67	85	119	**30**	24	37
	genome B	73	75	73	107	75	71	74	121	51	**31**	8
	genome D	84	87	76	104	76	69	77	126	56	55	**30**

*Numbers in boldness on the main diagonal denote the paralogous blocks within a genome, numbers above the diagonal denote the orthologous blocks between two genomes, while the numbers below the diagonal denote the out-paralogous blocks between two genomes*.

**Table 2:** Number of paralogous and orthologous gene pairs within and among the selected Poaceae genomes.

**Species**		***Oryza sativa***	***Aegilops tauschii***	***Triticum urartu***	***Hordeum vulgare***	***Setaria italica***	***Sorghum bicolor***	***Brachypodium distachyon***	***Zea mays***	***Triticum aestivum***		
										**Genome A**	**Genome B**	**Genome D**
*Oryza sativa*		**3,249**	,5000	4,591	6,642	16,330	14,564	15,427	14,678	7,998	7,998	8,388
*Aegilops tauschii*		1,714	**9,22**	4,295	6,871	4,920	4,384	4,782	4,971	6,157	6,026	6,448
*Triticum urartu*		1,213	623	**1,631**	6,379	4,674	4,284	5,062	4,889	4,859	4,929	4,582
*Hordeum vulgare*		2,211	1,419	1,296	**2,927**	8,245	7,201	8,516	8,047	9,044	8,679	8,875
*Setaria italica*		10,062	1,562	1,071	2,958	**3,634**	15,441	15,002	15,663	8,631	8,778	9,699
*Sorghum bicolor*		10,548	1,536	1,135	3,273	5,839	**4,223**	13,634	15,842	7,727	8,294	8,470
*Brachypodium distachyon*		10,280	1,363	1,052	3,129	4,544	4,660	**4,427**	14,371	9,205	9,934	10,163
*Zea mays*		8,370	1,672	1,311	3,130	4,635	4,816	3,851	**6,614**	9,282	9,359	9,605
*Triticum aestivum*	genome A	2,483	1,133	988	2,034	4,527	4,479	3,980	4,679	**2,852**	8,197	8,255
	genome B	2,484	1,151	1,023	2,040	4,508	4,953	5,135	3,712	1,447	**2,589**	7,993
	genome D	2,597	1,221	1,094	2,079	5,239	5,391	6,003	4,940	1,530	1,536	**2,778**

*Numbers in boldness on the main diagonal denote the paralogous gene pairs within a genome, numbers above the diagonal denote the orthologous gene pairs between two genomes, while numbers below the diagonal denote the out-paralogous gene pairs between two genomes*.

**Table 3:** Number of paralogous and orthologous genes within and among the selected Poaceae genomes.

**Species**		***Oryza sativa***	***Aegilops tauschii***	***Triticum urartu***	***Hordeum vulgare***	***Setaria italica***	***Sorghum bicolor***	***Brachypodium distachyon***	***Zea mays***	***Triticum aestivum***		
										**Genome A**	**Genome B**	**Genome D**
*Os*		**5,082**	4,723/4,653	4,461/4,510	6,472/6,467	14,545/13,284	12,601/12,697	13,530/12,892	12,989/11,124	7,742/7,800	7,742/7,800	8,113/8,176
		**13.2**	8.5/12.1	18.5/11.7	16.3/16.8	41.4/34.4	37.1/32.9	53.1/33.4	40.0/28.8	33.5/20.2	32.8/20.2	34.8/21.2
*Ae*		1,571/1,622	**1,614**	3,545/3,893	6,238/6,291	4,615/4,495	4,125/4,158	4,554/4,359	4,639/4,038	5,443/5,604	5,384/5,530	5,690/5,867
		4.1/2.9	**5.0**	14.7/7.0	15.7/11.3	13.1/8.1	12.1/7.5	17.9/7.8	14.3/7.3	23.5/10.1	22.8/9.9	24.4/10.5
*Tu*		1,175/1,143	597/570	**2,204**	5,863/5,664	4,576/4,144	4,172/3,956	4,945/4,432	4,713/3,839	4,623/4,558	4,650/4,558	4,198/4,318
		3.0/4.7	1.1/2.4	**14.2**	14.8/23.4	13.0/17.1	12.3/16.4	19.4/18.3	14.5/15.9	20.0/18.9	19.7/18.9	18.0/17.9
*Hv*		2,079/2,098	1,290/1,268	1,176/1,214	**4,381**	7,731/7,251	6,753/6,647	8,128/7,521	7,547/6,288	8,270/8,439	8,061/8,272	8,234/8,468
		5.4/5.3	2.3/3.2	4.9/3.1	**11.7**	22.0/18.2	19.9/16.7	31.9/18.9	23.3/15.8	35.7/21.2	34.1/20.8	35.3/21.3
*Si*		8,128/8,496	1,454/1,463	993/1,055	2,522/2,757	**5,735**	14,253/15,044	13,416/13,490	14,089/12,769	7,394/8,233	7,562/8,319	7,986/9,026
		21.1/24.2	2.6/4.2	4.1/3.0	6.3/7.8	**16.3**	41.9/42.8	52.6/38.4	43.4/36.3	32.0/23.4	32.0/23.7	34.3/25.7
*Sb*		8,933/8,744	1,459/1,436	1,069/1,115	2,794/3,081	5,475/5,335	**6,028**	12,986/12,222	14,904/12,713	7,048/7,320	7,447/7,687	7,582/7,854
		23.1/25.7	2.6/4.2	4.4/3.3	7.0/9.1	15.6/15.7	**17.7**	50.9/35.9	45.9/37.4	30.5/21.5	31.5/22.6	32.5/23.1
*Bd*		8,532/8,550	1,245/1,278	976/1,024	2,791/2,932	4,040/4,297	4,007/4,400	**5,469**	12,615/11,315	7,893/8,692	8,242/9,138	8,447/9,338
		22.1/33.5	2.2/5.0	4.0/4.0	7.0/11.5	11.5/16.8	11.8/17.3	**21.4**	38.9/44.4	34.1/34.1	34.9/35.8	36.2/36.6
*Zm*		6,153/6,966	1,365/1,562	1,068/1,282	2,381/2,930	3,738/4,263	3,898/4,471	3,142/3,574	**9,196**	6,995/8,607	7,163/8,726	7,352/8,921
		15.9/21.5	2.5/4.8	4.4/4.0	6.0/9.0	10.6/13.1	11.5/13.8	12.3/11.0	**28.3**	30.2/26.5	30.3/26.9	31.5/27.5
*Ta*	A	2,390/2,363	1,061/1,023	929/925	1,897/1,827	4,149/3,784	4,140/3,907	3,664/3,526	4,183/3,553	**4,026**	7,817/7,796	7,812/7,798
		6.2/10.2	1.9/4.4	3.8/4.0	4.8/7.9	11.8/16.4	12.2/16.9	14.4/15.2	12.9/15.4	**17.4**	33.1/33.7	33.5/33.7
	B	2,400/2,357	1,076/1,009	962/957	1,901/1,833	4,228/3,832	4,577/4,338	4,613/4,202	3,450/2,927	1,369/1,359	**3,749**	7,660/7,633
		6.2/10.0	1.9/4.3	4.0/4.1	4.8/7.8	12.0/16.2	13.5/18.4	18.1/17.8	10.6/12.4	5.9/5.7	**15.9**	32.9/32.3
	D	2,518/2,480	1,140/1,078	1,017/1,012	1,935/1,875	4,803/4,378	4,957/4,721	5,429/5,044	4,400/3,766	1,458/1,459	1,449/1,458	**3,975**
		6.5/10.6	2.0/4.6	4.2/4.3	4.9/8.0	13.7/18.8	14.6/20.3	21.3/21.6	13.6/16.2	6.3/6.3	6.1/6.3	**17.1**

*Numbers in boldness on the main diagonal denote the paralogous genes (upper) and percentages of them in total genes (below) within a genome, numbers above the diagonal denote the orthologous genes (upper two numbers) and percentages of them (lower two numbers) in two compared genomes (horizontal/vertical), while numbers below the diagonal denote the corresponding items of the out-paralogous genes between two compared genomes*.

Considering the intergenomic homology, we found that common wheat A, B, and D subgenomes had 96–615 orthologous blocks containing 4,582–10,163 collinear gene pairs, and 67–126 out-paralogous blocks containing 988–6,003 collinear gene pairs, as compared with other genomes (Tables 1, 2). Compared with the other grasses, orthologous regions or genes found between the common wheat A, B, and D subgenomes are more than those found between each of them and each of *A. tauschii* and *T. urartu*, but similar to those found between each of them and other grasses (Table 2). Between the subgenomes A, B, and D, there were, respectively, 24 A–B, 37 A–D, and 8 B–D orthologous regions containing 8,197, 8,255, and 7,993 collinear gene pairs, accounting for ~60% of the predicted genes. Besides, there were 51 A–B, 56 A–D, and 55 B–D (out) paralogs produced by the GCT containing 1,447, 1,530, and 1,536 collinear gene pairs, accounting for ~6% of total genes in each subgenome, respectively, which are two times fewer than the number of outparalogs between barley, rice, sorghum, Brachpodium, and maize (Table 3). Especially, each of the wheat three subgenomes has preserved ~40% more outpraralogs with other grasses, excluding *A. tauschii*, and *T. urartu*, than between any two of wheat subgenomes (Table 3). The far fewer outparalogous collinear genes found between these subgenomes of common wheat points to possible genome fractionation after its origination through extra polyploidizations.

With respect to the alignment of those grasses that were not common wheat, we have updated he inference reported previously (Wang X. et al., 2015b) based on the latest versions of the genome data available (Middleton et al., 2014; Du et al., 2017; Mascher et al., 2017; Zimin et al., 2017).

We used barley as a reference to construct the alignment table of common wheat and its diploid relatives (Figure 2). Here, we found, with the available genome sequences, barley had a better homology with each of the three wheat subgenomes than with each of two diploid wheat genomes, and had a similar level of homology to that with Brachypodium (Table 3). The orthologous collinear genes between barley and each wheat subgenome are ~25% more than between barley and each of diploid relatives.

**Figure 3:**
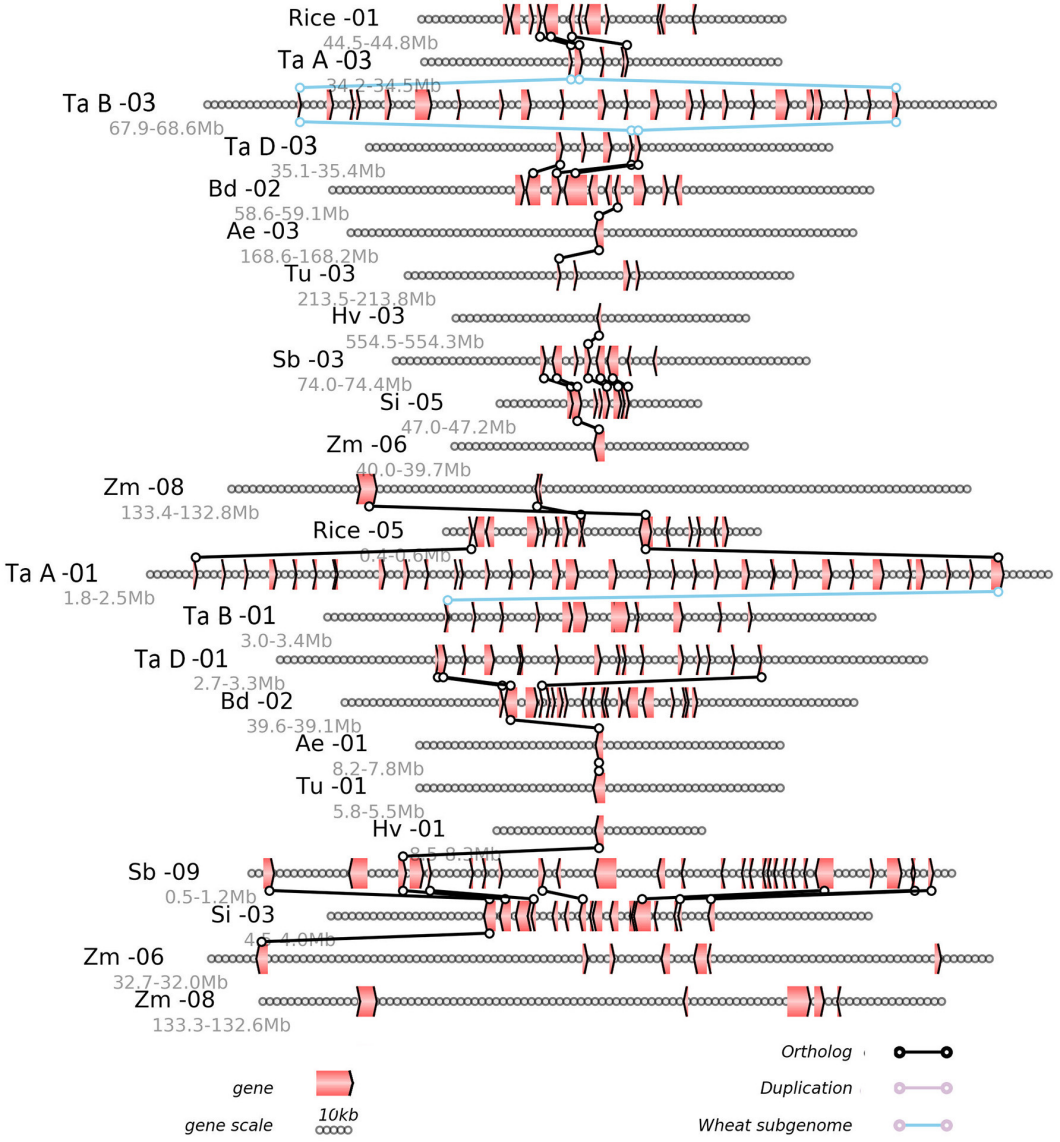
Alignment of the local regions sharing homology. Ae, Aegilops tauschii; Bd, *Brachypodium distachyon*; Si, *Setaria italica;* Hv, *Hordeum vulgare*; Rice, *Oryza sativa*; Sb, *Sorghum bicolor*; Tu, *Triticum urartu*; Zm, *Zea mays*; Ta A, genome A of *Triticum aestivum*; Ta B, genome B of *Triticum aestivum*; Ta D, genome D of *Triticum aestivum*. Genes are shown with pointed boxes to indicate their transcriptional direction. Homologous genes between neighboring chromosomes (indicated by the straight lines) are linked to lines with circles at their ends.

Any local region of the genome alignment can be linearly displayed to view the details of aligned genes, as well as the gene losses or translocations found there. The alignment of local regions often revealed the large-scale gene losses after the GCT and the lineage-specific events after their divergence (Figure 3). Using as reference the rice chromosomes 1 and 5, which were produced by the GCT, we displayed the alignment of a region from 44.5 to 44.8 Mb on rice chromosome 1, along with its corresponding regions from all other (sub) genomes (Figure 3). For example, this region in rice chromosome 1 was orthologous to those regions from 34.2 to 34.5 Mb on chromosome 3 of the common-wheat subgenome A, for which three collinear genes were shared. The region also shared orthology with a region from 67.9 to 68.6 Mb on chromosome 3 of genome B, and a region from 34.2 to 34.5 Mb on chromosome 3 of genome A. We also found a paralogous region with two collinear genes located on rice chromosome 5, which shared orthology with chromosome 1 of the subgenome A of common wheat. For the most part, however, the local alignment figure reveals only a few collinear genes, thus suggesting the occurrence of widespread gene losses or removal from their ancestral location.

### Chromosome reorganization

The alignment of chromosomes illustrates neatly how chromosome reorganization may have occurred after its divergence with rice, which was supposed to have preserved much of the ancestral grass karyotype after the GCT. Judging by the inner half set of circles of global alignment (Figure 1), rice chromosome 1 was fully preserved in chromosome 3 of wheat, and its close relatives, including barley. A similar phenomenon is evident for many other rice chromosomes except chromosome 3, which was split into parts to form wheat chromosome 4 and 5.

## Discussion

The comparative analysis of homology within and between the 9 grasses enhances our understanding of the evolution of grasses. Nearly 30 million years after a whole-genome duplication event ~100 million years ago (Paterson et al., 2004; Wang et al., 2005), the common ancestor of sorghum, maize, and foxtail millet were separated from the common ancestor of wheat, rice, barley, and purple false brome (Hilu, 2004).

Our study is a considerable expansion of prior published work inferring gene collinearity (Salse et al., 2008; Murat et al., 2010, 2014; Wang X. et al., 2015a). Here, we added common wheat to the execution of a multiple-cereal genome alignment. As an important group of Poaceae plants, wheat crops experienced both the GCT, and the hybridization that occurred between the wheat subgenomes. The common wheat plant of today is a result of the sequential hybridizations of wheat genome A with genome B, followed by hybridization with genome D. Thus, it includes three subgenomes; this has made the present wheat genome structure much complex. Besides, we included the most updated genome assemblies, and/or annotations of other genomes in the present analysis.

We constructed a collinearity table of genes that were hierarchically associated with the polyploidizations and speciations during the evolution of grasses. Doing so provides an important comparative genomics platform to support future related research in grasses. The gene collinearity dataset for these studied grass genomes is valuable in several ways. Firstly, researchers can use it to gain new insight into the chromosome segments of interest to find out how their genes were affected by genomic changes. This is possible because the collinear genes work as anchors to help locate specific DNA changes in the intragenic regions and in the regulatory cis-elements. Secondly, the collinear genes displayed in the alignment table can be used to construct phylogenetic trees for later use in sophisticated evolutionary analyses (Supplementary Table 2). Specifically, the information provided by our study clarifies when and how these genes originated, and diverged, thus providing robust data to support the pursuit of their functional innovation, especially for cases of duplicated genes. For plants, such duplicated genes are currently a “hotspot” of research activity (Innan, 2009; Mun et al., 2009; Wang et al., 2009; Wang H. et al., 2015). Thirdly, the new data we provide here may help resolve problematic trees that have been constructed to date. Plant genes evolve at very divergent rates and using this information in isolation might lead to wrong phylogenetic trees that fail to reflect the true relationships among plant taxa (Wang and Paterson, 2013). Here, by contrast, gene collinearity clearly displays the actual relationships among genes to better help construct a correct phylogenetic tree, which forms the sound basis of any evolutionary, and functional analysis.

Characterization of the homology within common wheat, and between it and the other grasses, shows that fewer outparalogous but similar orthologous collinear genes occur within common wheat or between its three subgenomes and other grasses, excluding *A. tauschii*, and *T. urartu*. A similar orthology between them and between each of them with some other grasses may mean that genome fractionation may have resulted small pieces of translocated regions, resulting in a higher effect eroding outparalogy but not orthology, smaller pieces of the latter being able to be inferred through gene collinearity. This result may be partially explained by still incomplete genome assembly (so far). However, considering that the wheat genome was sequenced and assembled later than its two diploid relatives—using similar and even better technology—it is quite plausible that much genome instability, and fractionation have happened since the formation of the ancestral hexaploid only ~10,000 years ago (Mayer et al., 2014). Though it was viewed as an allopolyploid, homoeologous chromosomes might have been much diverged before the formation of the hexaploid, illegitimate recombination should have occurred to accumulate considerable effect over time. This inference is tenable when considering that the GCT was previously proposed as an allopolyploid (Murat et al., 2014), and that it may have resulted in non-negligible homoeologous recombination (Wang et al., 2009). A pair of the GCT homoeologous of the grasses, rice chromosomes 11 and 12 (and their respective orthologs in other grasses), have been illegitimately recombining with each another at one of their terminal regions for millions of years and this process is still on-going in the *Oryza* species (Jacquemin et al., 2011; Wang et al., 2011). There is solid evidence suggesting that homoeologous recombination has resulted in large-scale gene losses, possibly by incurring breakages in the DNA double helix that has led to gene conversion, and thus it perhaps represents a mechanism of transmitting information between homoeologous genes (Gaeta and Chris Pires, 2010; Chen, 2013). More evidence of homoeologous exchanges can be found with *Brassica napus* (Cai et al., 2014), which is an allotetraploid of similar time of origination (Chalhoub et al., 2014). It seems that the homoeologous recombination between the common wheat subgenomes has been extensive and remains ongoing. The cumulative effect of this process may have contributed to wheat's domestication and the innovation of key biological functions, all of which invites further research in conjunction with population genomic data.

## Author contributions

XW conceived the study and led the research. JW and SS implemented and coordinated the analyses. SS, JW, JY, FM, RX, LW, ZW, WG, XL, YLi, YLiu, and NY performed the analysis. XW, SS, and JW wrote the paper.

### Conflict of interest statement

The authors declare that the research was conducted in the absence of any commercial or financial relationships that could be construed as a potential conflict of interest.
