# Supplementary material for: Alignment of Common Wheat and Other Grass Genomes Establishes a Comparative Genomics Research Platform
Source: Front Plant Sci. 2017 Aug 30;8:1480. doi: 10.3389/fpls.2017.01480 (PMC5582351; doi:10.3389/fpls.2017.01480)
Supplement: Supplementary file 1 [file Table1.DOCX]

**Supplementary Table 1. Information of genome data.**

| **Species name** | **Common name** | **Version** | **Genes** | **Genes**  **anchored on chr** | **Data source** | **Reference** |
| --- | --- | --- | --- | --- | --- | --- |
| *Oryza sativa* | Rice | IGDBV2 | 38714 | 38600 | MBK  (https://www.mbkbase.org) | Du H, Yu Y, Ma Y, et al, 2017^[1]^ |
| *Aegilops tauschii* | Wheat D | Aet_MR_1.0 | 55633 | 32061 | NCBI  (https://www.ncbi.nlm.nih.gov) | Zimin A V, Puiu D, Luo M C, et al, 2017^[2]^ |
| *Triticum aestivum* | Common wheat | TGACv1.36 | 104272 | genomeA 23139 | Ensembl Plants  (http://plants.ensembl.org/index.html) | Clavijo B J, Venturini L, Schudoma C, et al, 2017^[3]^ |
|  |  |  |  | genomeB 23628 |  |  |
|  |  |  |  | genomeD 23306 |  |  |
| *Triticum urartu* | Wheat A | ASM34745v1 | 24169 | 15503 | NCBI  (https://www.ncbi.nlm.nih.gov) | Middleton C P, Senerchia N, Stein N, et al, 2014^[4]^ |
| *Brachypodium distachyon* | Purple false brome | v3.1 | 31694 | 25502 | JGI  (https://phytozome.jgi.doe.gov/pz/portal.html) | International Brachypodium Initiative, 2010^[5]^ |
| *Hordeum vulgare* | Barley | PGSB_r1 | 39734 | 37577 | IBSC  (http://webblast.ipk-gatersleben.de/barley_ibsc) | Mascher M, Gundlach H, Himmelbach A, et al, 2017^[6]^ |
| *Setaria italica* | Foxtail millet | v2.2 | 40599 | 35158 | JGI  (https://phytozome.jgi.doe.gov/pz/portal. html) | Zhang G, Liu X, Quan Z, et al, 2012^[7]^ |
| *Sorghum bicolor* | Sorghum | v3.1 | 34496 | 34008 | JGI  (https://phytozome.jgi.doe.gov/pz/portal.html) | Paterson A H, Bowers J E, Bruggmann R, et al, 2009^[8]^ |
| *Zea mays* | Maize | Ensembl-18 | 32504 | 32441 | JGI  (https://phytozome.jgi.doe.gov/pz/portal.html) | Schnable P S, Ware D, Fulton R S, et al, 2009^[9]^ |

[1] Du H, Yu Y, Ma Y, et al. Sequencing and de novo assembly of a near complete indica rice genome[J]. Nature Communications, 2017, 8: 15324.

[2] Zimin A V, Puiu D, Luo M C, et al. Hybrid assembly of the large and highly repetitive genome of Aegilops tauschii, a progenitor of bread wheat, with the mega-reads algorithm[J]. Genome Research, 2017.

[3] Clavijo B J, Venturini L, Schudoma C, et al. An improved assembly and annotation of the allohexaploid wheat genome identifies complete families of agronomic genes and provides genomic evidence for chromosomal translocations[J]. Genome research, 2017, 27(5): 885-896.

[4] Middleton C P, Senerchia N, Stein N, et al. Sequencing of Chloroplast Genomes from Wheat, Barley, Rye and Their Relatives Provides a Detailed Insight into the Evolution of the Triticeae Tribe[J]. Plos One, 2014, 9(3): e85761.

[5] International Brachypodium I. Genome sequencing and analysis of the model grass Brachypodium distachyon[J]. Nature, 2010, 463(7282): 763-8.

[6] Mascher M, Gundlach H, Himmelbach A, et al. A chromosome conformation capture ordered sequence of the barley genome[J]. Nature, 2017, 544(7651): 427-433.

[7] Zhang G, Liu X, Quan Z, et al. Genome sequence of foxtail millet (Setaria italica) provides insights into grass evolution and biofuel potential[J]. Nat Biotechnol, 2012, 30(6): 549-54.

[8] Paterson A H, Bowers J E, Bruggmann R, et al. The Sorghum bicolor genome and the diversification of grasses[J]. Nature, 2009, 457(7229): 551-6.

[9] Schnable P S, Ware D, Fulton R S, et al. The B73 maize genome: complexity, diversity, and dynamics[J]. Science, 2009, 326(5956): 1112-5.
