# Supplementary material for: Alignment of Common Wheat and Other Grass Genomes Establishes a Comparative Genomics Research Platform
Source: Front Plant Sci. 2017 Aug 30;8:1480. doi: 10.3389/fpls.2017.01480 (PMC5582351; doi:10.3389/fpls.2017.01480)
Supplement: Supplementary file 3 [file Table3.DOCX]

**Supplementary Table 3. Number of homologous blocks within and among selected Poaceae genome.**

| **Homologous Blocks within and among genome** | **Block length** | | | |  |  |
| --- | --- | --- | --- | --- | --- | --- |
|  | **> 4** | **> 10** | **> 20** | **> 50** | **LDB^a^** | **LDBC^b^** |
| *Oryza sativa* | 3415/326 | 1844/43 | 1450/13 | 1320/10 | 285 | OS01-OS05 |
| *Brachypodium distachyon* | 6160/735 | 2595/82 | 1942/28 | 1483/13 | 253 | BD02-BD02 |
| *Setaria italic* | 8575/491 | 6340/85 | 5691/34 | 5213/18 | 798 | SI02-SI02 |
| *Sorghum bicolor* | 6265/684 | 2964/84 | 2312/32 | 1899/19 | 291 | SB03-SB09 |
| *Zea mays* | 8231/788 | 4851/160 | 3706/74 | 2070/19 | 257 | ZM02-ZM07 |
| *Hordeum vulgare L.* | 3307/527 | 826/43 | 459/12 | 243/4 | 73 | HV02-HV06 |
| *Aegilops tauschii* | 6375/239 | 5181/15 | 5070/7 | 5070/7 | 1253 | AE01-AE01 |
| *Triticum urartu* | 2554/453 | 373/24 | 100/3 | -/- | 38 | TU05-TU05 |
| *genome A of Triticum aestivum* | 4054/619 | 1070/38 | 810/17 | 418/5 | 139 | TAA01-TAA03 |
| *genome B of Triticum aestivum* | 3806/584 | 986/38 | 651/13 | 512/8 | 79 | TAB06-TAB07 |
| *genome D of Triticum aestivum* | 3969/602 | 988/34 | 745/15 | 379/4 | 152 | TAD01-TAD03 |
| *T. aestivum A genome vs T. aestivum B genome* | 12107/967 | 7531/50 | 7238/26 | 6859/15 | 1157 | TAA02-TAB02 |
| *T. aestivum A genome vs T. aestivum D genome* | 12790/1068 | 7748/56 | 7392/27 | 7203/21 | 1129 | TAA02-TAD02 |
| *T. aestivum B genome vs T. aestivum D genome* | 12738/1038 | 7824/49 | 7491/22 | 7301/16 | 1126 | TAB02-TAD02 |
| *O. sativa vs T. aestivum A genome* | 18451/1691 | 10379/123 | 9505/55 | 9023/40 | 860 | OS01-TAA03 |
| *O. sativa vs T. aestivum B genome* | 18129/1665 | 10102/111 | 9423/59 | 8656/35 | 916 | OS01-TAB03 |
| *O. sativa vs T. aestivum D genome* | 18960/1791 | 10398/127 | 9527/56 | 8946/37 | 873 | OS01-TAD03 |
| *B. distachyon vs*  *T. aestivum A genome* | 21018/2257 | 10081/154 | 8921/66 | 7947/35 | 874 | BD02-TAA03 |
| *B. distachyon vs*  *T. aestivum B genome* | 20634/2202 | 9959/133 | 9019/61 | 8120/33 | 940 | BD02-TAB03 |
| *B. distachyon vs*  *T. aestivum D genome* | 21489/2321 | 10293/144 | 9238/63 | 8356/35 | 958 | BD02-TAD03 |
| *S. italic vs T. aestivum A genome* | 16066/1406 | 9458/141 | 8616/78 | 7505/42 | 675 | SI05-TAA03 |
| *S. italic vs T. aestivum B genome* | 16056/1418 | 9454/138 | 8564/71 | 7597/39 | 730 | SI05-TAB03 |
| *S. italic vs T. aestivum D genome* | 16732/1503 | 9769/148 | 8850/75 | 7738/40 | 757 | SI05-TAD03 |
| *S. bicolor vs T. aestivum A genome* | 20273/2134 | 9907/126 | 9061/61 | 8530/42 | 835 | SB03-TAA03 |
| *S. bicolor vs T. aestivum B genome* | 19779/2069 | 9806/122 | 9075/61 | 8303/35 | 890 | SB03-TAB03 |
| *S. bicolor vs T. aestivum D genome* | 21110/2268 | 10166/136 | 9221/58 | 8591/38 | 918 | SB03-TAD03 |
| *Z. mays vs T. aestivum A genome* | 22020/2366 | 11015/232 | 9527/120 | 7500/55 | 519 | ZM03-TAA03 |
| *Z. mays vs T. aestivum B genome* | 21099/2249 | 10668/220 | 9247/113 | 7385/53 | 558 | ZM03-TAB03 |
| *Z. mays vs T. aestivum D genome* | 22450/2448 | 10965/218 | 9601/114 | 7659/53 | 578 | ZM03-TAD03 |
| *H. vulgare vs*  *T. aestivum A genome* | 18944/2179 | 8552/163 | 7642/93 | 6069/44 | 593 | HV03-TAA03 |
| *H. vulgare vs*  *T. aestivum B genome* | 18027/2009 | 8545/165 | 7496/81 | 6215/42 | 607 | HV03-TAB03 |
| *H. vulgare vs*  *T. aestivum D genome* | 19192/2187 | 8885/164 | 7890/86 | 6463/41 | 618 | HV03-TAD03 |
| *A. tauschii vs*  *T. aestivum A genome* | 14692/2153 | 4061/124 | 3038/40 | 2483/21 | 259 | AE07-TAA07 |
| *A. tauschii vs*  *T. aestivum B genome* | 13946/2033 | 3986/126 | 2911/40 | 2335/19 | 343 | AE03-TAB03 |
| *A. tauschii vs*  *T. aestivum D genome* | 15326/2219 | 4470/135 | 3278/36 | 2755/19 | 323 | AE04-TAD04 |
| *T. urartu vs T. aestivum A genome* | 15948/1960 | 6185/85 | 5475/25 | 5122/14 | 854 | TU02-TAA02 |
| *T. urartu vs T. aestivum B genome* | 15985/2122 | 5368/93 | 4568/28 | 4151/15 | 582 | TU03-TAB03 |
| *T. urartu vs T. aestivum D genome* | 16465/2177 | 5521/99 | 4704/33 | 4102/13 | 722 | TU02-TAD02 |

^a^number of collinear gene pairs reside in longest duplicated block (LDB); ^b^LDB on chromosomes (LDBC).
